# Comment on “Is Artificial Intelligence (AI) currently able to provide evidence-based scientific responses on methods that can improve the outcomes of embryo transfers? No.”

**DOI:** 10.5935/1518-0557.20240079

**Published:** 2024

**Authors:** Hinpetch Daungsupawong, Viroj Wiwanitkit

**Affiliations:** 1 Private Academic Consultant, Phonhong, Lao People’s Democratic Republic; 2 Saveetha Medical College and Hospital, Saveetha Institute of Medical and Technical Sciences, Saveetha University, Chennai, Tamil Nadu, India

Dear Editor, we would like to share ideas on “Is Artificial Intelligence (AI) currently able to provide evidence-based scientific responses on methods that can improve the outcomes of embryo transfers? No” (Kolokythas & Dahan, 2024). This study offers a valuable look into the usage of AI chatbots to give evidence-based therapeutic recommendations for infertility therapy, specifically embryo transfer outcomes. However, various limitations, both methodological and scope-related, should be rigorously considered. First, the dependence on only nine chatbots raises questions about the representativeness and dependability of the responses. The AI models utilized are constrained by their underlying data and programming, which might induce bias and inconsistency. Furthermore, the nature of the prompts, such as asking the chatbots to write an essay, does not guarantee that the findings adhere to current clinical principles or best practices in reproductive medicine. This constraint emphasizes the need for a broader strategy, including a larger range of AI platforms and even using other question styles.

Another major restriction is the focus on only 43 suggestions over 19 categories, with only a tiny proportion of them being evidence-based. This demonstrates a substantial gap in chatbots’ capacity to separate serious scientific evidence from anecdotal or unverified data. The abundance of divergent and controversial recommendations calls into question chatbots’ dependability in offering clinical recommendations. Future research should investigate the logic behind these responses, specifically how the algorithms prioritize and pick information. This could provide details about the AI training process and inherent biases. Furthermore, knowing the limitations of training datasets and algorithms could aid in the development of more robust AI systems that are more consistent with evidence-based medical procedures.

Furthermore, while the study identified crucial activities facilitated by chatbots, it also emphasizes an important factor: the ever-changing developments in medical knowledge and practice. The medical sector is continually changing, especially in specialty fields like reproductive medicine. Further inquiry is required to determine how frequently these AI models are updated with the most recent research findings and healthcare guidelines. New research could look at longitudinal evaluations of AI-generated suggestions over time to see if they are consistent with current clinical standards and beneficial in increasing embryo transfer outcomes when new evidence becomes available.

In terms of innovation, future research could consider incorporating a multidisciplinary strategy that blends AI with expert reviews from reproductive specialists, which could aid in the creation of AI systems that offer therapeutic recommendations based on existing evidence-based practices. Furthermore, combining patient and physician feedback into AI model training could lead to more user-centered outcomes. Finally, the goal should not only be to evaluate the correctness of AI-generated recommendations, but also to expand their usability as decision support tools in clinical settings, particularly at high-priority stages like embryo transfer. By addressing these challenges, the integration of AI into infertility treatment can be more successfully used to improve patient results.

## AI declaration

The author use language editing computational tool in preparation of the article.
